# The Compressed Vocabulary of Microbial Life

**DOI:** 10.3389/fmicb.2021.655990

**Published:** 2021-07-07

**Authors:** Gustavo Caetano-Anollés

**Affiliations:** Evolutionary Bioinformatics Laboratory, Department of Crop Sciences, and C. R. Woese Institute for Genomic Biology, University of Illinois, Urbana, IL, United States

**Keywords:** evolution, Heaps’ law, Menzerath-Altmann’s law, molecular functions, persistence triangle, semantics, Zipf’s law, word clouds

## Abstract

Communication is an undisputed central activity of life that requires an evolving molecular language. It conveys meaning through messages and vocabularies. Here, I explore the existence of a growing vocabulary in the molecules and molecular functions of the microbial world. There are clear correspondences between the lexicon, syntax, semantics, and pragmatics of language organization and the module, structure, function, and fitness paradigms of molecular biology. These correspondences are constrained by universal laws and engineering principles. Macromolecular structure, for example, follows quantitative linguistic patterns arising from statistical laws that are likely universal, including the Zipf’s law, a special case of the scale-free distribution, the Heaps’ law describing sublinear growth typical of economies of scales, and the Menzerath–Altmann’s law, which imposes size-dependent patterns of decreasing returns. Trade-off solutions between principles of economy, flexibility, and robustness define a “triangle of persistence” describing the impact of the environment on a biological system. The pragmatic landscape of the triangle interfaces with the syntax and semantics of molecular languages, which together with comparative and evolutionary genomic data can explain global patterns of diversification of cellular life. The vocabularies of proteins (proteomes) and functions (functionomes) revealed a significant universal lexical core supporting a universal common ancestor, an ancestral evolutionary link between Bacteria and Eukarya, and distinct reductive evolutionary strategies of language compression in Archaea and Bacteria. A “causal” word cloud strategy inspired by the dependency grammar paradigm used in catenae unfolded the evolution of lexical units associated with Gene Ontology terms at different levels of ontological abstraction. While Archaea holds the smallest, oldest, and most homogeneous vocabulary of all superkingdoms, Bacteria heterogeneously apportions a more complex vocabulary, and Eukarya pushes functional innovation through mechanisms of flexibility and robustness.

## Introduction

“*The place where I come from is a small town*,

*They think so small, they use small words*

*But not me, I’m smarter than that, I worked it out*

*I’ve been stretching my mouth, to let those big words come right out*”

—Peter Gabriel, “Big time” from his 1986 album “So”

Communication is an undisputed central activity of life that emerges from interpreting *signs*, signals that convey functional information in biological organization (Witzany, 2014, 2016). This activity of conveying meaning (*see* Box 1 for definitions) is constrained by the evolution and hierarchy of molecular and cellular structure and by dissipation of energy and information (Caetano-Anollés et al., 2010, 2017; Caetano-Anollés, 2021). The genetic code, for example, constitutes a remarkable natural language that links genetics to molecular functions (Eigen, 1971). Other languages of this type are much less understood. Here, I explore the vocabularies of molecules and associated functions responsible for the complex molecular and cellular structure of the organisms of Bacteria and Archaea. First, I discuss evidence supporting active communication that follows language rules and laws in the molecular repertoires of proteins (proteomes) and their associated molecular functions (functionomes). Second, I discuss how language laws are constrained by the engineering of the emerging biological systems and trade-off solutions between economy, flexibility, and robustness. Third, I focus on the evolution of molecular and functional vocabularies and how they reveal illuminating patterns of molecular origin and diversification that are consistent with engineering trade-offs. These trade-offs define constraints that lead to language compression (Box 1), herein interpreted as economy-driven optimization favored by the stable, rapid, and efficient propagation/processing of biological information (Krakauer, 2002). My goal is to use the communication paradigm to help dissect fundamental similarities and differences in the systems biology of the two microbial superkingdoms.

**Box 1. A brief primer of linguistic nomenclature.**

*Communication:* The use of messages to convey meaning.

*Compression:* A process of conveying the same message with a smaller number of signs.

*Dominance:* Relationship of primitiveness that forces dependence between lexical units.

*Grammar:* Rewriting rules specifying language; logical forms, abstractions, or functions that govern the structure and meaning of signs.

*Language:* A formal symbolic system of complex communication that uses signs and rules to communicate meaning.

*Lexeme:* Elementary unit of lexical meaning.

*Meaning:* The relationship between signs and what they intend, express, or signify.

*Message:* A discrete unit of communication of information.

*Metaphor:* The transfer of meaning from one word, image, or abstraction to another to uncover hidden and cognitively important similarities. From the *Greek*, μεταφ*o*ρα´ (*metaphorá*), transfer, μεταφε´ρω (*metapheró*), to carry over.

*Pragmatics:* Context-dependent relation of sign and sign user according to rules that govern successful or unsuccessful communication.

*Precedence:* Relation of order or adjacency of semantically related words in a lexical chain (or operators in computer programming) that provides cohesion in meaning (or mathematical expression).

*Semantics:* Meaning of signs and their combinations at different hierarchical levels of the message.

*Sign:* A semiotic entity that communicates a meaning and is causally related to an object through interpretation (e.g., word or subword drawn from some alphabet).

*Syntax:* The systematic combination of the inventory of signs according to semiotic rules of grammar.

*Vocabulary:* The set of signs used to communicate meaning (its lexicon).

## The Linguistics of Proteomes and Functionomes

**FIGURE 1:**
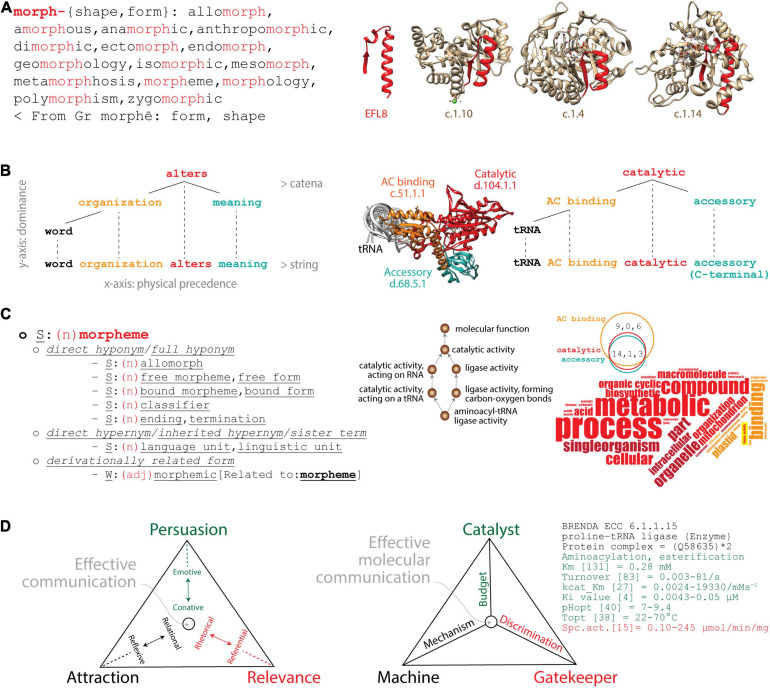
Analogies between linguistics and molecular biology. **(A)** Lexicon–Module correspondence: Morphemes combine to form words (left) as “elementary functional loops” (EFLs) combine to form structural domains in proteins (right). The morpheme “morph” is a lexical unit (highlighted in red) of cognate words with common origin. The list of cognates retrieved from the Cognātarium (http://www.cognatarium.com/cognatarium/) includes the word “morpheme.” EFL8 (highlighted in red) is a structural unit of β/α-barrel domains with common evolutionary origin, including the catalytic domain of RubisCo (c.1.14), Earth’s major carbon-fixing enzyme (Caetano-Anollés, 2017). **(B)** Syntax–Structure correspondence: Words combine to form language expressions (left) as protein structural domains combine to form protein structures (right). The dependency grammar of a clause can be described with an ordered tree and catenae reflecting actual word order. The syntactic hypothesis is constructed top-to-bottom to provide a meaning to the message. Similarly, a dependency molecular grammar can also be constructed for the prolyl-tRNA synthetase (ProRS) multidomain enzyme (PDB entry 1 h 4 s) with its catalytic, anticodon (AC) binding and accessory structural domains. The age of domains is traced on an atomic model of the enzyme, red being ancient and turquoise recent. The tRNA is viewed coaxially from its anticodon (AC)-binding arm and serves as a point of reference. **(C)** Semantics–Function correspondence: Hyponyms and hypernyms describe semantic relationships as Gene Ontology (GO)-based lexical and conceptual semantic statements describe biological functions. The semantic field of “morpheme” (noun) can be described with sets of cognitive synonyms (synsets) expressing individual concepts and visualized interlinked by means of conceptual-semantic and lexical relations using the WordNet^®^ lexical database (https://wordnet.princeton.edu). Similarly, GO terms associated with the “aminoacyl-tRNA ligase activity” of the ProRS structural domains form a network of functional associations. A word cloud of GO terms extracted from the *Superfamily* database (https://supfam.mrc-lmb.cam.ac.uk/SUPERFAMILY/) describes domain-associated biological processes (*bp*), molecular functions (*mf*), and cellular compartments (*cc*) (depicted as vertical, horizontal, and oblique words, respectively); the Venn diagram shows how *bp*, *mf*, and *cc* terms (in that order) are shared by domains. Red terms describe properties of the catalytic domain (which are common to all domains), and orange terms describe properties unique to the AC-binding domain. Highlighted in yellow is “ligase activity,” the only *mf* term describing the catalytic core and the main enzymatic activity of the enzyme. Darker shades are terms that do not apply directly to the respective domains. Note how the accessory domain does not have unique terms, since there are no words colored turquoise. **(D)** Pragmatics–Fitness correspondence: A triangle of effective communication describes how context contributes to meaning as a triangle of effective molecular communication describes the fitness parameters of a molecular system. Triangles showcase trade-off solutions of Persuasion, Relevance, and Attraction imposed by linguistic factors and functions (in parentheses): sender (emotive) and receiver (conative), context (referential) and message (rhetorical), contact (relational) and code (reflexive). Pairs of these functions push effective communication toward individual vertices of the triangle to maximize fitness. The triangle describing effective molecular communication of the ProRS enzyme (EC 6.1.1.15) pushes matter–energy budget toward the Catalyst vertex (Persuasion), discrimination toward the Gatekeeper vertex (Relevance), and mechanism toward the Machine vertex (Attraction). The Brenda repository of enzyme information (https://www.brenda-enzymes.org) provides parameters (see list as examples) that can inform about trade-offs.

Communication occurs when one entity conveys meaning to another through “messages” composed of mutually understood signs (e.g., words), the collective of which form vocabularies. Communication can be studied at four levels: lexicon, syntax, semantics, and pragmatics (Box 1). The lexicon is a message “wordbook,” a catalog of signs used by higher levels of language organization. Syntax embodies the set of rules, principles, and processes that govern the combination of those signs in messages. Semantics focuses on the meaning of messages in vocabularies and grammars. Pragmatics studies how the transmission of meaning is affected by the context of the message, including the intention of the entities that are communicating. There are clear correspondences between the *Lexicon ⇒ Syntax ⇒ Semantics ⇒ Pragmatics* progression of language organization and the *Module ⇒ Structure ⇒ Function ⇒ Fitness* paradigms of biology (modified from Searls, 2001, 2002; Figure 1):

*(i) Lexicon–Module correspondence.* Elementary units of lexical meaning (*lexemes*, Box 1) are very much like molecular *modules*, sets of integrated parts that cooperate to perform a biological task (Figure 1A). Modules are generally recognized by the property of *modularity*, the degree to which component parts of any system can be separated and rearranged. Morphemes, for example, are the smallest grammatical units of a language. They can form words by themselves or they can combine with other morphemes to generate an inventory of words with a common origin. Conversely, loop structures (∼25–30 amino acid residues long) that are stabilized by the formation of van der Waals locks are recurrent in proteins (Berezovsky and Trifonov, 2001). Some of these structural units are “elementary functional loops” (EFLs) that hold molecular functions are evolutionarily conserved and often combine in groups of 2–3 to form folded structural domains with active, binding, or regulatory sites (Goncearenco and Berezovsky, 2015). Figure 1A illustrates how the glycine-rich EFL8 motif prototype (highlighted in red) that appeared for the first time ∼3.5 billion years (Gy) ago (Aziz et al., 2016) was recruited into the aldolase (superfamily c.1.10) and the FMN-linked oxidoreductase (c.1.4) domains ∼3.3 Gy ago before the oxygenation of the planet and then into the C-terminal domain of the large subunit of RuBisCo (c.1.14) ∼1.5 Gy ago (Caetano-Anollés, 2017). A lexicon also exists in structural domains, which combine to form “architectures” in multidomain proteins (Bashton and Chothia, 2007; Wang and Caetano-Anollés, 2009; Aziz and Caetano-Anollés, 2021), or in proteins, which assemble by self-interaction into quaternary protein complexes (Levy et al., 2008). This modularity that exists in molecular structure paraphrases that of the English language (Figure 1A).

*(ii) Syntax–Structure correspondence.* The syntax of natural languages involves the combination of words to form meaningful expressions such as sentences (textual units that include clauses). This combinatorics can be studied with “*dependency grammar*” constructs supported by a modern syntactic theory that uses graph–theoretical strategies to study dependency relations between linguistic units (Tesnière, 1959). For example, the *catena* (Latin for “chain”) is a unit of syntax that projects to a string of words (O’Grady, 1998). Given the expression “*word organization alters meaning*” (Figure 1B), the verb “*alters*” is placed at the center of the clause structure and its dependencies (one-to-one word correspondences) are built around it from top to bottom as the clause unfolds from left to right in a string of words (Osborne et al., 2011, 2012). Similarly, a dependency grammar exists in an aminoacyl-tRNA synthetase (aaRS) enzyme, which is responsible for the specificity of the genetic code (Figure 1B). The catalytic aminoacylating domain with the oldest and most central functional role (appearing ∼3.5 Gy ago; Caetano-Anollés et al., 2013) is placed at the center of the multidomain enzymatic architecture. Dependencies are then built top-to-bottom with more evolutionarily derived domains that enhance the specificities of the initial catalytic core, starting with the anticodon (AC)-binding domain (∼3.1 Gy ago) and an accessory domain (1.6 Gy ago) as the polypeptide string unfolds from the amino to the carboxy terminus. In both the language and molecular cases, the *catena* is a combination of lexical units that is continuous on the vertical dimension of dominance, with the most dominant (or primitive) lexical unit on the top of the “hierarchical order,” while the *string* is continuous on the horizontal dimension of precedence (Caetano-Anollés, 2021). See Box 1 for definitions. Projection edges connect the lexical tree of the catena to the string to form a complete network structure.

*(iii) Semantics–Function correspondence.* Semantics dissects the meaning of linguistic expressions at the level of either words or subwords (*lexical semantics*) or arguments (*conceptual semantics*) (Devitt and Hanley, 2006). Lexical semantics explores the syntax–semantics interface with, for example, semantic networks that relate concepts to each other using *synonymy* (sameness in meaning), *hyponymy* (subordination), and *hypernymy* (superordination). To illustrate, hypernyms are higher-level terms that group lower-level hyponyms in a transitive relation of the “is a” type. A direct hyponym of the word “morpheme” (minimal meaningful language unit) retrieved from the WordNet^®^ database is “allomorph” (variant phonological representation of a morpheme) (Figure 1C). In other words, an allomorph is a morpheme. Conversely, a direct hypernym of morpheme is “language unit” (one of the natural units into which linguistic messages can be analyzed). Similar semantic relations have been developed by the Gene Ontology (GO) database to standardize the functional annotation of gene products with a vocabulary of ontological terms describing the biological processes (*bp*), molecular functions (*mf*), and cellular components (*cc*) of viruses and cellular organisms (Ashburner et al., 2000). GO *mf* terms define *activities* (actions characterizing molecular agents), *bp* terms define *events* (objects and their properties manifesting in time), and *cc* terms define *components* (parts of a biological whole and “places” where activities and events occur). GO *mf*, *bp*, and *cc* terms form directed acyclic graph (DAG) structures, complex tree-like networks in which child terms can be connected with multiple parents and lower DAG levels represent more specialized functional annotations (Harris et al., 2004; Shegogue and Zheng, 2005). To illustrate, “ligase activity, forming carbon–oxygen bonds” (GO:0016875) describing the central enzymatic activity of the aaRS enzyme (aminoacylation of the acceptor arm of the tRNA) establishes an “is a” relationship to the higher-level term “ligase activity” (GO:0016874), while “aminoacyl-tRNA ligase activity” (GO:0004812) is its more specialized child term (Figure 1C). The three aaRS domains share 14, 1, and 3 *bp*, *mf*, and *cc* terms, respectively. Their functions can be described with a word cloud that holds the “ligase activity” *mf* at its core.

*(iv) Pragmatics–Fitness correspondence.* Pragmatics explores how context influences the meaning of messages (Korta and Perry, 2015). A triangle of effective communication, for example, describes how pairs of linguistic functions push communication toward individual vertices of a triangle that maximizes the trade-off solutions of *Attraction*, *Persuasion*, and *Relevance* (Figure 1D). Inspired by the “organon model” (Bühler, 1934) and the six “constitutive factors” of Jakobson (1960), the triangle places main goals at its vertices, each defining pairs of opposing forces (Caetano-Anollés, 2021). To drive communication toward the *Persuasion* vertex, the sender must “persuade” (influence) the receiver through an emotive–conative dynamic relationship. To push communication toward the *Relevance* vertex, context and message, and their respective referential and rhetorical functions, are used to prompt an impactful “action” that is relevant and faithful to the message. Finally, relational and reflexive functions are used to attract attention to the subject of the message and drive communication toward the *Attraction* vertex. To illustrate, narrations in epic poetry, emotive expressions of lyric poetry, and perspectives in scientific discourse preferentially push communication toward the Relevance, Persuasion, and Attraction vertices, respectively. The triangle embodies a “fitness” function for the factors, functions, and principles of language that are optimized when the trade-off solutions find a balance. It is therefore fitting that the meaning of the word “pragmatics” (*Greek*, πραγματικó*ς*) is “*fit for action*” (n., pragma = deed act; v., prasso = be successful). The sphere in the triangle of Figure 1D represents a single point (or “quasispecies cloud”) in the trade-off space of communication performance. These points locate in Pareto fronts, boundaries in trade-off multidimensional spaces that provide best-fitness solutions with known geometries (plane in the three-dimensional performance space of the triangle; Sheftel et al., 2013). A similar triangle of effective communication exists in molecular biology that maximizes the fitness of genotypes through phenotypes and the potential of persistence of molecular communication in changing environments (Figure 1D). In communication parlance, senders and receivers are components of the biomolecular systems that exchange messages in the form or matter, energy, and information. An aaRS enzyme, for example, maximizes effective molecular communication by acting as “catalyst,” “gatekeeper,” and “machine” in a triangle of communication (Caetano-Anollés, 2021). The three goals are driven by budget (economy), discrimination (robustness), and mechanism (flexibility). An appropriate matter–energy budget ensures effective catalysis involving, for example, the optimization of substrate and enzyme concentrations, temperature, and pH. This pushes the catalytic Persuasion vertex by influencing the matter–energy flow. The aaRS enzyme must also discriminate its substrate by, for example, making use of appropriate editing, AC-binding, and accessory functions needed for conformational proofreading. This ensures the molecule acts as gatekeeper of catalysis. This pushes the discriminatory Relevance vertex by ensuring the faithful transmission of the catalytic message. Finally, the enzyme must also provide a mechanism for the enzymatic machine to function that optimizes potential energy of the molecular system, movement, and information and energy transfer. This pushes the mechanistic Attraction vertex by accreting the molecular components and contacts for stable and reliable communication.

When describing these correspondences, I use a number of analogies between linguistics and molecular biology as *metaphors*, symbolic representations of some abstraction (Box 1), to open significant discussion of how genomics and systems biology can help understand biological complexity. The makeup of proteomes and functionomes provides clues to the different levels of complexity of molecular language organization of individual organisms, or collectively, of superkingdoms of life. The comparative and evolutionary analysis of information in completely sequenced genomes, which enabled the exploration of the origin, evolution, and structure of the protein world (Caetano-Anollés et al., 2009), can help us dissect how language laws are constrained by trade-off solutions.

## Language Laws Are Constrained by the Engineering of Biological Systems

**FIGURE 2:**
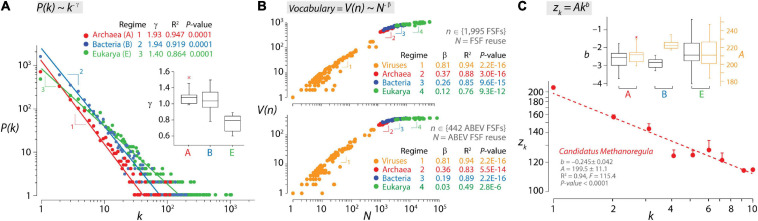
Laws of language describe growth of structural domains in proteomes. **(A)** The double logarithmic plot describes the relationship between the frequency of individual protein folds, *P(k*), occurring *k* times in the proteomes of Archaea (A), Bacteria (B), and Eukarya (E), which follows a Zipf’s law. The relationship between frequency and occurrence was fitted to a straight line that drops off sharply with different regimes (indicated by numbers) for the proteomes of Archaea/Bacteria and for those of Eukarya. Additional power law statistics for individual regimes [α = 2.000 ≫ 1; Kolmogorov–Smirnov (KS) fit = 0.169–0.328 ≪ 1, KS *p*-value = 0.132–0.174 ≥ 0.05] supported the data being drawn from a power law distribution. Regimes are also evident for individual proteomes in boxplots of parameters of exponential decay γ. Data from Caetano-Anollés and Caetano-Anollés (2003). **(B)** The double logarithmic plots describe a four-regime Heaps’ law of vocabulary growth for structural domains defined at fold superfamily (FSF) level for all 1,995 FSFs (top plot) or only for the 442 FSFs that are common to all superkingdoms and viruses (ABEV FSFs; bottom plot). Archaea, Bacteria, Eukarya, and viruses have their own power law regime of allometric scaling. *V(n)* represents vocabulary size (use of FSFs) and *N* represents database size (reuse of FSFs in each of the 368 proteomes analyzed). Data from Nasir et al. (2017). **(C)** The Menzerath–Altmann (MA) law of language in the structural domains of an archaeal proteome. A log–log plot shows how the average length of structural domains in a protein (*z*_*k*_) decreases with the number (*k*) of structural domains it contains for the entire proteome of *Candidatus Methanoregula*. Vertical bars represent the standard error of the means. The two-parameter power law formulation of the MA law facilitates fitting parameters in log–log plots to a straight line. The hatched regression line indicates the weighted linear fitting. Boxplots describe decay parameters *b* and intercepts *A* (when *k* = 1) for the proteomes of the three superkingdoms. Data from Shahzad et al. (2015).

It can be argued that molecular language follows quantitative linguistic patterns that arise from statistical laws that are universal (Caetano-Anollés et al., 2017; Caetano-Anollés, 2021). These patterns include probability distributions and functional and developmental type laws that are often interrelated (Altmann and Gerlach, 2016) and apply to biological makeup (Mazzolini et al., 2018a). Figure 2 shows three examples of how language laws manifest in the organization of the structural domains of proteins. The Zipf’s law is a probability distribution that describes the frequency of a word in a corpus of natural language as being inversely proportional to its rank in a word frequency table (Zipf, 1948). A mathematical formulation of the empirical law states that the numbers of words that are used (that occur) *k* times in a document decay according to *f(k)* = *Ck^–γ^*, where *C* is a normalizing constant and γ is the exponential parameter of decay (γ ≥ 1). The Zipf’s law is a special case of the scale-free distribution when *f(k)* is highly skewed and reflects a probability, with *P(k) ∼ k^–γ^*. The scale-free property follows power law distributions that are common properties of biological networks such as metabolic networks or protein–protein interaction networks (Strogatz, 2001). The presence of a power law distribution of genomic frequencies of genes or protein domains has been studied extensively, beginning with Huynen and van Nimwegen (1998), and may represent a general property of modular biological and technological systems with a multilayered dependency network (Pang and Maslov, 2013). However, power law derivations have yet to use the statistical class of “regularly varying distributions” to explain heavy-tailed phenomena likely present in biological distributions and networks and extreme value theory to accurately estimate γ tailed exponents (Voitalov et al., 2019). These rigorous regularly varying distributions formalize the traditional intuition behind the “*∼*” sign of the scale-free formula. At protein world level, we and others have shown that the frequency of structural domains in proteomes follows a Zipfian distribution at different levels of structural abstraction (from the fold to the family level; Qian et al., 2001; Caetano-Anollés and Caetano-Anollés, 2003; Lagomarsino et al., 2009). The analysis of proteomes, individually or pooled by an organismal domain of life, showed that archaeal and bacterial organisms shared scaling regimes that were steeper than those of the unicellular and multicellular organisms of Eukarya (Figure 2A). These divergent Archaea/Bacteria and Eukarya regimes match those for English (γ = 1.9) and Chinese (γ = 1.5), respectively (Li et al., 2016), suggesting that there are profound differences in the dynamics of vocabulary construction manifesting in both proteomes and natural languages. Note, however, that matching regime γ tailed exponents is non-trivial. For example, ranked frequency distributions of Chinese texts may be better fitted by stretched exponential functions than by power laws (Deng et al., 2014). In addition, languages show “size-effects” generally present in the tails of frequency distributions that are dependent on the size of text corpora. In turn, genome and proteome sizes are delimited by evolutionary constraints rather than database size. Finally, Zipfian distributions depend on the definition of Zipfian units of the multilevel structured systems being studied (see an example in music; Perotti and Billoni, 2020), which are vastly different in languages and protein structure. Differences in vocabulary dynamics also materialize when studying the growth and evolution of vocabularies through correlations of language properties with either time or accumulating innovation (Nasir et al., 2017). In fact, a stochastic duplication/innovation model explains the concurrent emergence of the Zipfian distribution and a Heaps’ law (Lagomarsino et al., 2009) through dependency structures in the form of directed networks describing component systems (Mazzolini et al., 2018b). The Heaps’ law is a developmental type that, for example, describes how the vocabulary (number of different words) *V* in a document or corpora scales with the database of words *N* measured in number of words (text length) according to the general expression *V(n)* = *KN*^β^, with K and β being empirically determined parameters (Herdan, 1964; Heaps, 1978). The signature of the law is sublinear growth (β < 1) typical of “*economies of scale*” that show increasingly weaker returns for new vocabulary innovations when systems expand into the adjacent possible (Tria et al., 2018). For human languages, “kernel” words appearing with high frequency in vocabularies are constant over centuries (Ferrer i Cancho and Solé, 2001), and their growth can be described in terms of database size (Petersen et al., 2012; Gerlach and Altmann, 2013). Similarly, there is a “kernel” vocabulary of protein fold structures that is universally shared by superkingdoms and has been constant over billions of years of evolution (Caetano-Anollés et al., 2009; Caetano-Anollés et al., 2017). This property allows to construct a dynamic view of vocabulary innovation using the static view of the Heaps’ law. Figure 2B shows scatter log–log plots describing the relationship between the vocabulary of protein domains defined at fold superfamily (FSF) level of structural abstraction and the database of FSFs in proteomes for all 1,995 FSFs or only for the 442 FSFs that are common to all superkingdoms and viruses. The plots reveal a four-regime Heaps’ law of vocabulary growth describing a decreasing marginal need for new words and an evolutionary slowdown (cooling) that is similar to that of vocabularies for Indo-European, Chinese, Japanese, and Korean languages (Petersen et al., 2012; Lü et al., 2013; Li et al., 2016). The four individual regimes of allometric scaling corresponded to the proteomes of viruses, Archaea, Bacteria, and Eukarya, in that order (Figure 2B), showing increasing slowdown of vocabulary growth with β scaling exponents decreasing from 0.81 to 0.12–0.26. Plots suggest that viral proteomes use a very ancient kernel-like vocabulary with β exponents not far from the second regime of languages with limited vocabularies (β = 0.7–0.77; Petersen et al., 2012; Lü et al., 2013). This ancestral kernel is then expanded successively in evolution by vocabulary slowdowns, first in the proteomes of Archaea (matching slowdown of English text corpora, β = 0.4–0.7; Tria et al., 2014), then in Bacteria (matching the third regime of Chinese, β = 0.3; Petersen et al., 2012; Li et al., 2016), and finally (to an extreme) in Eukarya. Multi-regime vocabulary growth has been explained by a stochastic feedback model driven by two probabilities, one for the reuse of frequently used words (the kernel lexicon) and the other for the rise of word novelties (the unlimited lexicon) (Li et al., 2016), which then translates into a probability density function with at least two scaling regimes (Ferrer i Cancho and Solé, 2001). The final example of language materializing in molecular biology is a typical functional type law that links word frequency and word length in messages. This Menzerath–Altmann (MA) law can be summarized by the motto “*the greater the whole, the smaller its constituents*” (Menzerath, 1954), with wholes representing linguistic constructs (e.g., words) and constituents representing parts of those constructs (e.g., morphemes). The popular mathematical formulation of the law is the two-parameter version y*(x)* = *Ax*^*b*^, where *y(x)* is the length of the parts, *x* is the number of parts that make up the system (constructs of parts), and *A* and *b* are fitting parameters in log–log plots (Altmann, 1980). The MA law has been shown to apply to genomes (e.g., Ferrer-i-Cancho and Forns, 2010; Baixeries et al., 2012) and the organization of protein structural domains in proteomes (Shahzad et al., 2015). Figure 2C describes how the lengths of structural domains in the proteins of the archaeal *Candidatus Methanoregula* proteome decreased linearly with increasing domain number when data were fitted in log–log plots. The proteome is part of a dataset belonging to a highly curated analysis of structural domains in ∼3 million sequences of 745 proteomes (Wang et al., 2011b). Analysis of this dataset revealed that proteomes of the three cellular superkingdoms exhibited negative *b* slopes with high goodness of fit (*R*^2^ ranging from 0.85 to 1.00), with the broadest slope distribution present in Eukarya (ranging from –0.045 to –0.404) (Figure 2C). Steep slopes depict increased patterns of decreasing returns of domain length and weaker push toward molecular economy of domain organization. In turn, intercept *A* values reflect lengths of single-domain proteins (ranging from 183 to 247) and upper bounds (economic strata) for the size-dependent minimization principle of the MA law. To understand the drivers of the MA law, a persistence function *P* was formulated with two terms, one reflecting the matter–energy cost of adding domains and extending their length in proteins (*P*_*ME*_) and the other reflecting how information present in domain length and number influence the flexibility and robustness of the molecular system (*P*_*FR*_) (Shahzad et al., 2015). *P* defined a “triangle of persistence” that explained why steep slopes and larger intercepts favored flexibility and robustness, while shallow slopes and smaller intercepts favored economy of domain organization. Remarkably, while Archaea and Bacteria exhibited similar slopes, Archaea showed lower intercepts than Bacteria, highlighting the stronger push toward economy of Archaea at a lower economy stratum (Figure 2C). This was confirmed by a kingdom-level analysis, which revealed that proteomes of Archaea and fungi (and to a lesser degree plants) showed the largest push toward molecular economy, each at its own economic stratum (Shahzad et al., 2015). Thus, a complicated language-like behavior exists in protein structure that is constrained by universal laws and engineering principles.

## The Triangle of Biological Persistence Dissects Large Microbial Groups

**FIGURE 3:**
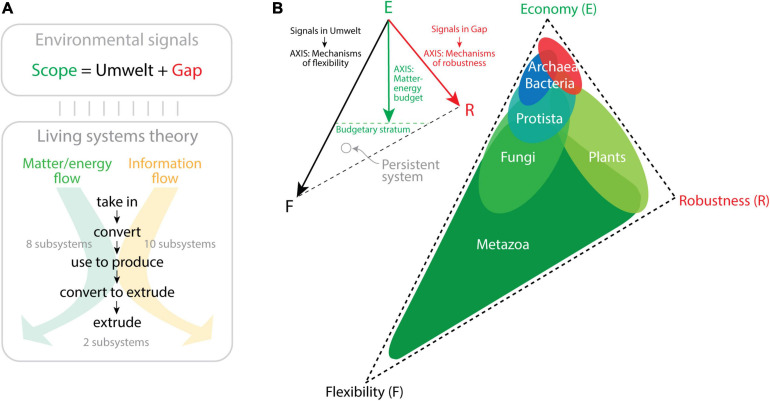
The triangle of biological persistence. **(A)** An open biological system is exposed to a wide range of environmental signals (the system’s Scope), which are either perceived (Umwelt) or not (Gap). These signals establish matter–energy and information flows that are processed by 20 critical subsystems enabling a multitude of processes and functions (described in Miller, 1978). Two kinds of processes that are interconnected can be established, “*communication*” processes that transform information from one state into another, and “*action*” processes, which move matter–energy in space–time. Both processes function by taking in and finally extruding matter–energy and information after processing through intermediate stages. **(B)** The triangle of persistence describes the space of evolutionary trade-off solutions among *economy*, *flexibility*, and *robustness.* The triangle is bounded by the three engineering strategies, with economy measured as matter–energy budget, flexibility measured as the number of signals an organism processes in its Umwelt through flexibility mechanisms, and robustness measured as the number of signals in the Gap and the mechanisms that confer robustness to those signals. The three axes define the placement of persistent systems (e.g., organisms) in the trade-off space. The location of organisms belonging to the six kingdoms of life are suggested in the triangle of the right based on ecological and molecular information (Yafremava et al., 2013; Shahzad et al., 2015; Mainzer et al., 2021).

Computers communicate across the Internet by transmitting data and delivering information without mistakes to the right computer address. In order to establish a digital communication system that is “persistent,” computer network participants must abide by a system of rules (a “*communication protocol*” such as the TCP/IP Internet protocol) defining the syntax, semantics, and pragmatics (synchronization) of computer names in a global namespace. The naming rules seek efficient and reliable exchange of information following a trilemma paradigm of compromises that ensures names are *memorable* (remembered without errors and with the least information), *secure* (protected from failure and attack), and decentralized (distributed to ensure control by many authorities). This constitutes Zooko’s *memorable–secure–decentralized* triangle (Wilcox-O’Hearn, 2001; Ferdous et al., 2009). Zooko’s triangle is reminiscent of the *persuasion–relevance*–*attraction* triangle of conversation and the *budget–discrimination–mechanism* triangle of molecular persistence I introduced in Figure 1D. Memorability in language usually involves semantic compression, an expression of economy of information (Chomsky, 1995; Sayood, 1996). Securing a naming system to avoid ambiguities or failure usually involves optimization to enhance communication robustness. A decentralized system allows versatility, which is an endowment of flexibility. Zooko’s triangle is also compatible with the general *economy–flexibility*–*robustness* triangle that arises from the persistence function of the MA law (Figure 2C). Note that this triangle of persistence was originally proposed by Yafremava et al. (2013) to describe the impact of the environment on a biological system. This theoretical framework, which is backed by significant ecological and molecular data, differs but was inspired by Grime’s *competitive–ruderal–stress-tolerant* triangle of plant life strategies (Grime, 1974). The persistence framework allowed to dissect the six major kingdoms of life in the triangle’s phase space of trade-off solutions (Figure 3). The main premise is that the environment constrains evolution of physiologies over initial and boundary conditions of the organismal system. Using Jacob von Uexküll’s organism-centric semiotic view of the environment (von Uexküll, 1909) and James G. Miller’s theory that organisms are open systems maintaining thermodynamically highly improbable states and operating with a set of 20 critical subsystems to dissipate matter–energy and information (Miller, 1978), the framework proposes two conceptual trios that map to each other, “scope/umwelt/gap” and “economy/flexibility/robustness” (Yafremava et al., 2013). von Uexkull defined “*umwelt*” (“the world around us”) as the totality of an organism’s perception, i.e., all signals (signs) that undergo sensory processing. However, not all received signals are processed by an organism. Those signals that are not perceived fall into the “gap” between the “umwelt” and the entirety of signals to which an organism is exposed during its lifetime, its “scope.” Conversely, the economy/flexibility/robustness trio embodies engineering principles associated with the processing of matter–energy and information that operate in the Miller’s critical subsystems (Mainzer et al., 2021). Most of these subsystems take in, convert, produce, and extrude matter/energy and information using a variety of dissipation pathways (Figure 3A). Economy reflects the budget of matter–energy costs of a system. Flexibility reflects structural and functional mechanisms requiring processing of information needed to respond and adapt to internal and external changes. Robustness embody mechanisms that use information to maintain structure and function in the face of environment-induced damage and change. Crucially, when a signal undergoes sensory processing, it is transformed by the internal organismal subsystems resulting in internal change or outward response. When the extent of that processing impacts adaptation and survival, then it is fixed in the lineage because it provides mechanisms of flexibility that promote persistence. Thus, “umwelt” maps to flexibility and defines a flexibility axis of the triangle (Figure 3B). This axis depicts the repertoire of flexibility mechanisms that are developed in response to the signals processed in the organism’s umwelt. Similarly, the robustness axis of the triangle measures the number of robustness mechanisms that arise from signals in the gap that challenge the structure and functioning of the organism. Note that gap signals are not processed, even if damaging. Organisms simply evolve robustness mechanisms to counteract their effect. Developing both flexibility and robustness is costly. For example, the flexible utilization of a wider panel of organic or inorganic substrates by a purple sulfur bacterium (Kumar et al., 2008) or the development of robust biology in a marine archaeal piezophile capable of withstanding life at ocean depths (barophyly) (Oger and Jebbar, 2010) demands significant resource investment of matter–energy. A distance from the economy vertex is therefore proportional to the matter–energy budget needed to support the flexibility and robustness mechanisms of the two initial axes. This establishes a matter–energy budgetary stratum. Organisms of the six kingdoms of life exhibit clear patterns of scope, budget, flexibility, and robustness derived from significant evidential support (e.g., speed, cell size, spatial range, life span, nutrition, molecular makeup; Yafremava et al., 2013). This information can be used to display the trade-offs between the three persistence strategies in the triangle (Figure 3B). The unicellular microbes of Archaea and Bacteria, which are the smallest and slowest cellular organisms and harbor the smallest genomic, proteomic, and functional repertoires, gravitate toward the economy corner of the triangle. Archaea favors robustness and Bacteria flexibility at their own budgetary strata. Their placement in the trade-off space likely results from reductive evolutionary pressures imposed by microbial size and viscous drag and their ability to compete for resources or withstand challenging environments. In contrast, the large and diverse number of flexibility mechanisms exhibited by the four kingdoms of Eukarya, many associated with multicellularity, pushes these organisms toward the flexibility corner. They overcome the limitations of microbial life at low Reynolds numbers and limited fluid inertia (Purcell, 1977), relieving reductive evolution pressures and facilitating expansive evolution. Note that the large number of flexibility mechanisms (which cost more matter–energy) and the advanced budgetary strata of Eukarya makes the triangle asymmetric with an extended flexibility leg. Metazoa are the most diverse and flexible organisms and are locked in a positive feedback loop toward flexibility. In turn, the robustness corner is populated by the ruderal-like Plant kingdom, while Fungi are more flexible but exhibit smaller budget, pushing the Economy strategy. Protista stand in the middle between prokaryotic organisms and the rest of Eukarya. Their sizes range from those typical of bacteria in picoplankton (e.g., *Ostreococcus tauri*) to those typical of plants in heterokonts such as giant kelp (*Macrocystis pyrifera*), which form giant forests in the Pacific Ocean.

Yafremava et al. (2013) proposed the existence of a “protistan saddle manifold” in the triangle that acted as origin of diversified life, with early viscosity-bound microbes undergoing reductive evolution toward the economy vertex. Given a molecular clock of folds (Wang et al., 2011a), these microbes diversified 2.9 Gy ago into Archaea by pushing robustness (e.g., in thermophilic and/or barophilic niches) and 2.1 Gy ago into Bacteria by pushing flexibility (e.g., in environments fostering competition and predation). Expansive eukaryotic evolution was then prompted by the rise of diversified Metazoans 1.6 Gy ago and then Fungi and Plants 1.4 Gy ago. The placement of the last universal common ancestor in the saddle manifold is consistent with the hypothesis that this cellular ancestor was a relatively large phagotrophic organism (Poole et al., 1998; Kurland et al., 2006).

## The Evolutionary Compression of Proteome Vocabularies

**FIGURE 4:**
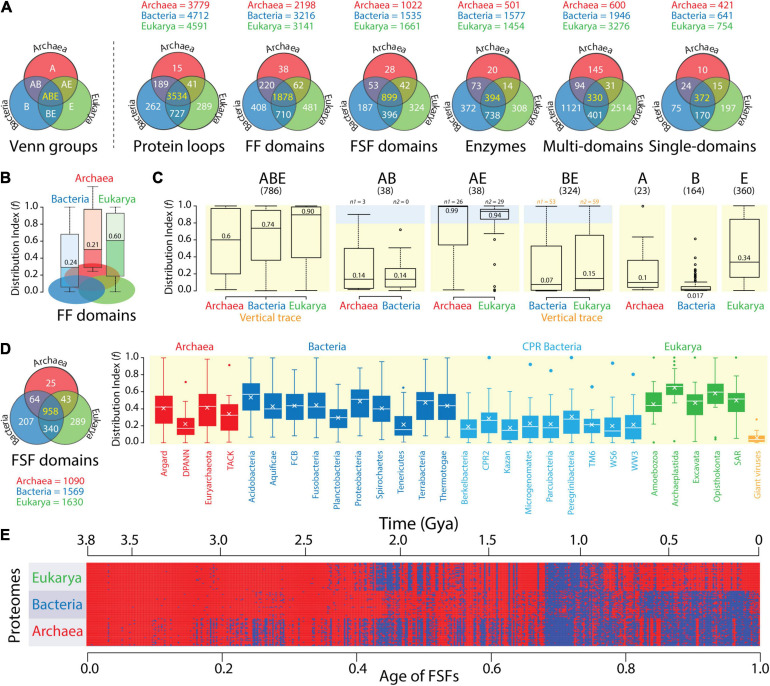
The compressed protein vocabularies of microbial domains. **(A)** Venn diagrams of molecular trait distributions in superkingdoms show vocabulary compression in Archaea. The Venn diagrams describe a censuses of protein loops (8,127 proteomes; Mughal and Caetano-Anollés, unpublished), structural domains defined at fold family (FF) and superfamily (FSF) levels of SCOP structural classification (8,127 proteomes; Mughal et al., 2020), enzyme domains (989 proteomes; Mughal and Caetano-Anollés, 2019), and multidomain and single-domain architectures of proteins in proteomes (266 proteomes; Wang and Caetano-Anollés, 2009). Numbers in the ABE Venn group describing the universal repertoires shared by superkingdoms Archaea (A), Bacteria (B), and Eukarya (E) are colored yellow. Those colored white are specific to individual or groups of superkingdoms. **(B)** A patchy distribution of traits shows a diverse vocabulary apportioned between the small proteomes of Bacteria. The spread of FF domain structures (*f*) in the proteomes of each superkingdom is shown in boxplot representations, with numbers indicating medians. Lower *f*-values indicate more patchy distributions. **(C)** Distribution patterns are confirmed by an analysis of Venn taxonomic groups. The spread of FSF domain structures (*f*) in the seven Venn taxonomic groups (panels ABE, AB, AE, BE, A, B, and E with their respective FSF numbers) of the proteomes of superkingdoms. Shaded regions indicate that FSFs were present in > 80% of proteomes (f > 0.8), and their numbers, n_1_ and n_2_. Numbers in boxplots indicate group medians. Numbers in orange suggest the strongest vertical evolutionary trace. Data taken from an analysis of 981 proteomes (Nasir and Caetano-Anollés, 2013). **(D)** FSF distributions in the proteomes of phyla confirm patchwork patterns spread throughout lineages of the microbial superkingdoms. Boxplots describe average *f*-values of FSFs belonging to the 15 metabolism subcategories and the five information subcategories of the SUPERFAMILY functional annotation scheme (http://supfam.org/SUPERFAMILY/function.html) mapped to phyla of the three superkingdoms and to NCLDV viruses, which are used as control. Crosses inside boxes describe mean values. The Venn diagram describes the vocabularies for FSF analyzed by Bokhari et al. (2020). **(E)** Phylogenetic data matrix with columns describing 442 ABE FSFs that are shared with viruses (detected in ∼11 million proteins and ordered according to evolutionary age) and rows describing a 368-proteome subset. The matrix given in heatmap format shows FSF presence (red) or absence (blue) in a proteome. Age is given as relative age from 0 (origin of proteins) to 1 (the present) and in real time (Gy ago) according to a molecular clock of protein folds. Data from Nasir and Caetano-Anollés (2015).

The triangle of persistence offers a pragmatic framework to explain the diversification and evolutionary history of cellular life. Conversely, comparative and evolutionary genomics provides the data and tools to understand how the lexicon, syntax, and semantics of molecular vocabularies interface with the pragmatic landscape of the triangle. An initial comparative analysis of proteomes at different levels of molecular complexity reveals that the push toward economy of Archaea and Bacteria results in two different strategies of language compression. Venn diagrams describing the diversity and distribution of molecular traits among Venn groups describing all possible relations between Archaea, Bacteria, and Eukarya confirm that there was significant proteome history in these diagrams (Figure 4). These molecular traits were surveyed in thousands of genomes. They included 5,057 protein loops (Mughal and Caetano-Anollés, unpublished), 3,797 structural domains defined at fold family (FF) level and 1,929 domains defined at FSF level (Mughal et al., 2020), 1,919 domains in metabolic enzymes (Mughal and Caetano-Anollés, 2019), and 4,636 multidomain and 863 single-domain protein architectures (Wang and Caetano-Anollés, 2009). Loops and domains stressed the lexicon of the protein vocabulary. Architectures stressed the syntax. Note that these molecular traits are encoded modules of complex component systems. Their numbers are expected to be proportional to genome size. For example, we found monotonic correlations between protein domain components and total in-frame coding capacity (i.e., coding sequence) (Wang et al., 2011b).

Archaea consistently harbored the smallest vocabularies (see trait numbers for superkingdoms above Venn diagrams) and the smallest number of Archaea-specific traits (the A Venn taxonomic group). In sharp contrast, Eukarya harbored on average 2.4-fold [±0.7 (SE); skewness = 1.22] larger vocabularies and 16-fold (±1.4; –0.18) larger numbers of superkingdom-specific traits than Archaea. The vocabularies and superkingdom-specific traits of Bacteria (B group) were comparable to those of Eukarya, on average only 1.1-fold (±0.1; 1.28) and 1.6-fold (±0.3; 0.31) smaller, respectively. The only exception were multidomain architectures, which were significantly numerous in Eukarya. All Venn diagrams showed a high representation of traits that were common to all three superkingdoms (the ABE Venn group), with percentages of these universal traits averaging 41.1% (±8.6%; –0.34) but ranging from 7.1% for multidomain architectures to 69.9% for protein loops (Figure 4A). The existence of a significant universal core strongly supports a common ancestor of diversified life. Similarly, the AB and AE Venn groups were 6-fold (±1.1; 0.39) and 19-fold (±6.8; 1.52) smaller in number than the BE group, respectively. This bias, which was consistent across genomic surveys, suggests an ancestral evolutionary link (a vertical trace) between Bacteria and Eukarya.

While Venn data suggest the protein vocabularies of Archaea are significantly compressed, similar patterns exhibited by Bacteria and Eukarya challenge the scaling relations of the domain probability distributions of Figure 2. A boxplot analysis of the distribution of traits in individual proteomes provided an explanation for this disparity (Figure 4B). The use of a *distribution index* (*f*) that effectively measures the popularity (spread) of structural domains (Wang et al., 2007) in individual proteomes revealed domains were sparsely distributed in both Archaea and Bacteria but less so in Eukarya. In the boxplots, the index describes the fraction of proteomes that use individual FFs on a relative 0–1 scale, with *f* = 0 and *f* = 1 reporting absence or presence of the FF trait in all proteomes, respectively. The lower median *f*-values of both Archaea and Bacteria, which were more than a third of those of Eukarya, indicated that both microbial superkingdoms had proteomes with significantly sparse distributions. Thus, the complex vocabularies of Bacteria that were evident in Figure 4A are differentially apportioned between proteomes of individual organisms.

Nasir and Caetano-Anollés (2013) extended this simple type of analysis to all Venn groups, enumerating how individual FSF domains distributed in the organisms of each superkingdom (Figure 4C). Boxplots for the 786 universal ABE FSFs revealed a progression of median *f*-values for Archaea (*f* = 0.6), Bacteria (0.74), and Eukarya (0.90). This result again supports the effect of evolutionary reductive forces acting on both microbial superkingdoms and the significant apportionment of FFs in proteomes. While ABE median *f*-values were among the highest of all Venn groups, only 17 FSFs were truly universal (*f* = 1) and only 245 FSFs had a nearly universal presence (*f* > 0.9). These sets are known to be enriched in metabolic functions (Kim and Caetano-Anollés, 2011). Thus, horizontal gene transfer, convergent evolution, and genome reduction likely facilitated the buildup of a patchwork of proteome makeup, while at the same time preserving a near-universal core that strengthens the hypothesis of life’s common ancestry. The strong vertical trace of the ABE taxonomic group was also present in the numerous BE group (324 FSFs). Despite its low median *f*-values (*f* < 0.15) and uniform spread, 53 and 59 FSFs were highly popular (*f* > 0.8) in Bacteria and Eukarya (light blue shaded boxplot region; Figure 4C), respectively. These numbers are larger than those of AE and AB groups, regardless of their *f*-distributions, confirming again the ancestral link between Bacteria and Eukarya. Finally, additional evidence for genome reduction and differential FSF spread in proteomes comes from the low *f*-values of the superkingdom-specific A, B, and E groups. In particular, only one of the 164 Bacteria-specific FSFs was present in > 50% of proteomes, highlighting the rarity of these structural innovations and suggesting independent acquisitions. Similar patterns were present in Archaea, but not in Eukarya, which showed wider FSF distributions. Thus, the lexica of Bacteria-specific vocabularies are rather similar to those of Eukarya but are apportioned heterogeneously between proteomes in ways resembling Archaea.

A more recent study of FSF distributions (Bokhari et al., 2020) highlights how the larger vocabulary of Bacteria was differentially apportioned between proteomes of different bacterial phyla as these were compared to those of Archaea and Eukarya (Figure 4D). Boxplots describe *f*-values for metabolism and information FSFs in 2,430 proteomes, including a large sampling of the highly reduced Candidate Phyla Radiation (CPR) bacterial group that lives in a wide range of environments. Median *f-*values of well-described bacteria phyla ranged from 0.16 to 0.57, those of CPR bacteria ranged from 0.14 to 0.28, those of archaeal phyla ranged from 0.17 to 0.43, and those of Eukarya ranged from 0.44 to 0.66. Thus, bacterial proteomes exhibit FSF patchworks with wider spread distributions than Archaea. In fact, the highly heterogeneous distribution of bacterial FSFs compared to those of the other two superkingdoms can be clearly visualized in a phylogenetic heat matrix of FSF occurrence in proteomes in which columns represent ABE FSFs shared with viruses and rows represent proteomes (Figure 4E). This heat matrix takes advantage of almost two decades of phylogenomic analyses of protein domain history (starting with Caetano-Anollés and Caetano-Anollés, 2003), which dates each and every protein domain defined at different levels of structural complexity. The time of origin (age) of each FSF was directly derived from a most-parsimonious phylogenomic tree of FSF domains that was reconstructed using the parsimony ratchet (methodology described, for example, in Nasir and Caetano-Anollés, 2015) and was rooted with Weston’s generality criterion (Caetano-Anollés et al., 2018). Age was expressed as a relative distance of internal nodes along branches of the tree from the root to each of its leaves and transformed to geological time scales using a clock of folds (Wang et al., 2011a). These types of trees describe the gradual evolution of the protein world by the slow accumulation of structural and functional innovations.

## The Evolutionary Compression of Functionome Vocabularies

**FIGURE 5:**
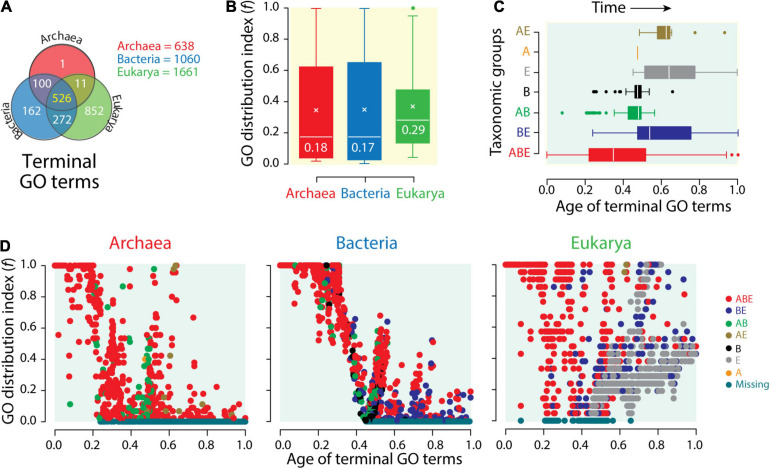
The compressed functional vocabularies of microbial domains. **(A)** Venn diagram showing the distribution of terminal Gene Ontology (GO) terms of molecular functions (GO_*TM*__*F*_) of the functionomes of superkingdoms among the seven Venn taxonomic groups (ABE, AB, AE, BE, A, B, and E). **(B)** The distribution of GO_*TM*__*F*_ terms among functionomes (*f*-index) of each superkingdom is displayed with boxplot representations. **(C)** Boxplots displaying the distribution of GO_*TM*__*F*_ terms corresponding to the seven Venn taxonomic groups along an evolutionary timeline of molecular functions, with time expressed as relative ages of GO_*TM*__*F*_ terms, 0 representing the first function and 1 the present. **(D)** Scatterplots displaying the distribution of GO_*TM*__*F*_ terms in functionomes of Archaea, Bacteria, and Eukarya as GO terms of molecular functions unfold in the evolutionary timeline.

While proteome vocabularies stress the lexicon and syntax of molecular languages, functionome vocabularies stress their semantics. A Venn diagram of terminal *mf* GO terms (GO_*TM*__*G*_) defining typical actions of molecular agents “associated” with gene products (Nasir and Caetano-Anollés, 2013) reveals the increasing compression of functional vocabularies in Bacteria and then Archaea. While the genomes of Eukarya encoded 1,661 GO_*TM*__*G*_ terms, those of Bacteria numbered 1,060 and those of Archaea only 638 (Figure 5A). The distributions of GO_*TM*__*G*_ terms in Venn groups showed similar patterns to those observed in proteomes (Figure 4A). A common core of 526 terms supported an ancient core of universal functions inherited from a common ancestor of life, and the 272 terms of the BE group gave again credence to a common origin of Bacteria and Eukarya. When compared to the 852 Eukarya-specific terms, the single Archaea-specific term and the 162 Bacteria-specific terms showcase significant compression of vocabulary innovations in these superkingdoms. Boxplots of *f*-values confirmed the significant biased spread of molecular functions that exists in the functionomes of microbial lineages (Figure 5B). While *f*-value distributions were significantly biased toward lower values, the median values for Bacteria and Archaea (*f* ∼0.18) were significantly lower than those for Eukarya (*f* = 0.29). Both Venn taxonomic groups and boxplot data support semantic compression in the language of microbial life.

An evolutionary view confirms the effect of reductive evolution on functionome vocabularies. Phylogenomic analyses define a natural history of molecular functions and an evolutionary timeline of innovations. This helps understand how the meaning of molecular messages has unfolded in evolution. Phylogenomic trees of molecular functions revealed the gradual evolutionary accretion of functional innovations at different levels of the DAG (Kim and Caetano-Anollés, 2010; Nasir and Caetano-Anollés, 2013; Nasir et al., 2014b; Kim et al., 2014; Koç and Caetano-Anollés, 2017). Inferences made from timelines of accretion were congruent with those made from the analyses of proteomes (Caetano-Anollés and Caetano-Anollés, 2003; Wang et al., 2007; Wang and Caetano-Anollés, 2009; Caetano-Anollés et al., 2011). Trees of functionomes confirmed patterns of diversification of superkingdoms made from trees of functions and identified thermophilic Archaea as the most ancient forms of cellular life (Kim et al., 2014; Nasir et al., 2014a). Timelines of GO_*TM*__*G*_ terms, for example, unfolded from the origin of functions (relative age of the founder term = 0) to the present (ages of the most recent terms = 1). The boxplots of GO_*TM*__*G*_ term distributions along the timeline of functions revealed a clear order of evolutionary appearance of Venn taxonomic groups, which followed the sequence ABE > BE > AB > B > E > A > AE (Figure 5C). The early appearance of the ABE and BE groups confirmed their ancient origin, which was intimated by comparative genomic analyses. The few AB GO_*TM*__*G*_ terms that appeared earlier than BE terms were identified as outliers and were likely candidates of horizontal gene transfer events occurring between Archaea and Bacteria later on in evolution (Nasir and Caetano-Anollés, 2013). One example was “penicillin binding (GO: 0008658),” which was universal in Bacteria but rare in Archaea. The late appearance of superkingdom-specific groups, starting with those from Bacteria and then from Eukarya and Archaea, supports inferences from comparative analyses and extensive phylogenomic studies of structural domains in proteomes [beginning with Wang et al. (2007)]. The late appearance of the AE group reinforces the common origin of Bacteria and Eukarya intimated by the early origin of the numerous BE group. Tracking *f*-values for individual GO_*TM*__*G*_ terms and superkingdoms along the timeline of functions showed the historical spread of functional innovations (Figure 5D). As expected, the most ancient terms were universal or widely distributed in functionomes. A total of 55 and 56 GO_*TM*__*G*_ terms in Archaea and Bacteria, respectively, had *f* = 1 and were both universal and the oldest in the timeline. Archaeal universal terms had ages older than 0.21. Bacterial universal terms were older than 0.32. In contrast, 125 GO_*TM*__*G*_ terms were universal in Eukarya and had ages between 0 and 0.8, almost appearing throughout the entire timeline. The high *f*-value of ancient and universal GO_*TM*__*G*_ terms decreased with passage of time as the relative age of terms decreased from origin of functions to the present. This is an expected result when newly appearing functions distribute “vertically” in the emergent lineages of an emerging tree of life. Later appearances restrict the occurrence of functional innovation to increasingly confined organismal clades. Remarkably, the expected drop of *f*-values with time unfolded differently when considering individual superkingdoms [as initially observed for protein folds by Wang et al. (2007)]. In Archaea, *f*-values dropped rapidly with time. In fact, the first complete functional loss (*f* = 0) occurred at an early age of 0.23. In Bacteria, the drop occurred later and more slowly with the first loss occurring at an age of 0.45. In Eukarya, *f*-values dropped similarly to Archaea. First losses were also comparable. When dismissing ancient functions impacted by horizontal transfer, such as “penicillin binding,” the first losses of terms in Eukarya occurred at times comparable to those of Archaea (terms had ages > 0.3). A contrasting pattern of increase in functional distribution with time is also evident in these plots. Many functions appearing at ages above 0.4 have high *f*-values, with some reaching universality. The contrasting pattern can only be explained by processes of horizontal exchange that spread functional innovation throughout emergent lineages. This “horizontal” opposing trend appears minimal in Bacteria and maximal in Eukarya, with Archaea in between.

The two clear patterns of functional diversification, together with traces of Venn taxonomic groups described in Figure 5D that are compatible with those of Figure 4, highlight two different evolutionary modes: a decreasing “losing” trend pushing *f*-indices to low values that is fostered by “vertical” dilution in emerging lineages and/or reductive evolution by functional loss and an “expansive” trend pushing *f*-indices to high values fostered by “horizontal” processes of recruitment, rearrangement, and recombination that enhance diversification (Wang et al., 2007). These horizontal processes were aided by a “big bang” of structural domain combination in proteins that occurred late in protein evolution and was particularly effective in Eukarya (Wang and Caetano-Anollés, 2009). To conclude, the data show that functionomes of Archaea and Eukarya were strongly constrained by the decreasing “losing” trend early in evolution. This initial trend occurred later in Bacteria. Horizontal exchange processes that started to occur later in the timeline at ages ∼0.5 enabled the expansive trend. This trend was maximal in Eukarya and minimal in Archaea. It occurred as clear bursts in Bacteria and to some extent in Archaea. The two strong opposing forces delimiting the repertoire of GO terms that exist in the functionomes of the three superkingdoms produced a historical “hourglass” of functional innovation that was magnified in Eukarya.

## Word Clouds Link Molecular Semantics and Pragmatics

Word clouds have been used as tagging systems for navigation and hypertext browsing of the World Wide Web. Founding examples include *Flickr*, *Del-icio-us*, and *Technorati*. While the benefit for navigation has been contested on network–theoretic and user–interface grounds (Helic et al., 2011), the word cloud continues to represent a useful device to distill the most relevant single words (tags) of a body of text. In social software applications, the frequency, significance, or categorization of words are aggregated over text and used to define the font size of words in the cloud. Highly weighted tags are often hyperlinked for website navigation.

Word clouds are being used successfully in molecular biology. For example, microarray and next-generation sequencing experiments define sets of genes that are relevant according to some criterion (e.g., differential gene expression, gene clustering). These gene lists can be functionally characterized using GO enrichment analysis methods such as those available in Database for Annotation, Visualization and Integrated Discovery (DAVID; Dennis et al., 2003), Gene Set Enrichment Analysis (GSEA; Subramanian et al., 2005), Babelomics (Al-Shahrour et al., 2005), WebGestalt (Zhang et al., 2005), or g:Profiler (Reimand et al., 2011). The mapping of genes to GO terms of the DAG is one to many. This makes the interpretations of enriched GO term lists difficult with standard approaches, including heatmaps, treemaps, network tracings, and scatterplots. Word clouds provide a solution to the representation of gene enrichment results. Several tools provide word cloud implementations in REVIGO (Supek et al., 2011), Cytoscape (Oesper et al., 2011), GeneCodis3 (Tabas-Madrid et al., 2012), Genes2WordsCloud (Baroukh et al., 2011), and GO summaries (Kolde and Vilo, 2015). For example, GO summaries implements word clouds from GO enrichment analyses and displays them associated with data from differential gene expression, clustering, or principal component analyses.

**FIGURE 6:**
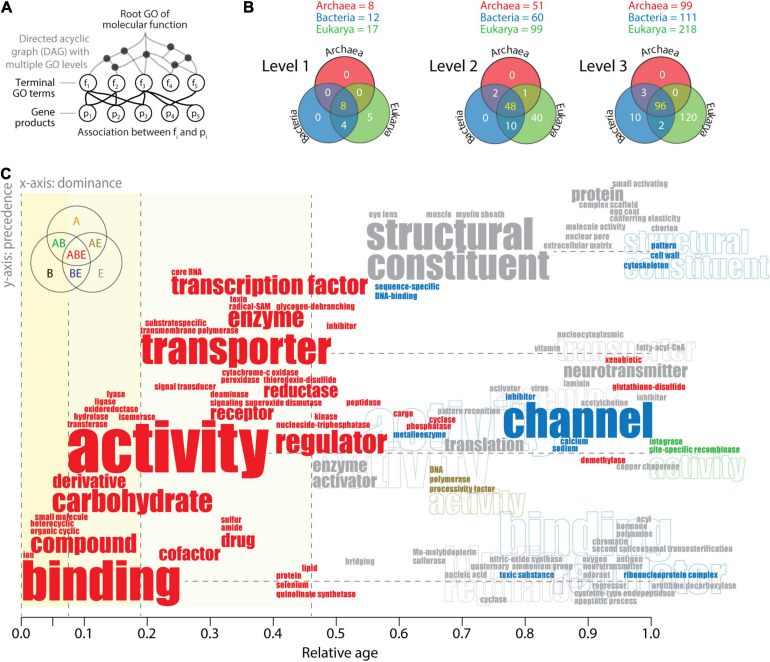
The history of level 2 Gene Ontology (GO) terms of molecular functions visualized with a causal word cloud. **(A)** The hierarchical structure of the DAG_*MF*_. GO terms can be categorized into GO levels by establishing *is_a* relationships with parental nodes and by including parental nodes into the level if they do not have child nodes (Kim and Caetano-Anollés, 2010). In addition, GO terms that appear as multiple instances in one or multiple levels are collapsed into a single term while excluding those that have *part_of* and *regulation* relationships. **(B)** Venn diagrams describe the distribution of GO terms between superkingdoms, when terms are defined at the three highest levels of the DAG_*MF*_. The list of GO terms can be found in Koç and Caetano-Anollés (2017) and term definitions in AmiGO 2 (http://amigo.geneontology.or/amigo/dd_browse) of the GO classification (Ashburner et al., 2000). **(C)** Word cloud of level 2 GO terms ordered according to evolutionary dominance (x-axis) and precedence in the lexical chain (y-axis). See text for description of the approach and algorithmic implementation. Word clouds were generated separately for each Venn taxonomic group of GO terms and then combined. Repeated lexical units are identified with outline fonts. Data from Koç and Caetano-Anollés (2017).

Word clouds can be useful tools for linking semantic and pragmatic views of biological languages. This can be accomplished at different hierarchical levels of ontological abstraction of the pyramid-like DAG. GO terms can be categorized into GO levels by establishing “annotations” of parental and child relationships (Figure 6A). A survey of the highest three GO levels mapped onto GO_*TM*__*F*_ terms in the functionomes of the three superkingdoms (Figure 6B) establishes Venn diagrams of GO term distributions (Kim and Caetano-Anollés, 2010; Koç and Caetano-Anollés, 2017). The existence of a central universal core and an ancestral evolutionary link between Bacteria and Eukarya is again strongly supported by Venn distributions. The numerous Eukarya-specific GO terms and the absence of GO terms specific to Archaea and Bacteria at levels 1 and 2, including the only 10 Bacteria-specific GO terms defined at level 3, support the strong reductive evolutionary force that operates on microbial superkingdoms. To make this push toward economy explicit, “causal word clouds” organized by evolutionary dominance and precedence were generated for each Venn taxonomic group at the three levels of the DAG_*MF*_. This approach takes advantage of the “dependency” grammar paradigm used in catenae (Figure 1B) in which “transitive” relationships of precedence that describe lexical adjacency are linked to relationships of dominance (primitiveness) that force dependence between lexical units (e.g., the implicit causal dominance of verbs over arguments; Hartshorne and Snedeker, 2013). Constraining the word cloud by dominance and precedence moves the dependency paradigm from the syntax–structure level to the semantics–function level. The x-axis of dominance orders the lexical units (words or word-chains) of GO terms according to their evolutionary age, and the y-axis of precedence orders the string of words defining the name of GO terms. Figure 6C illustrates the strategy with a “causal” word cloud for level 2 GO terms. Words are colored according to the Venn taxonomic group they belong. Their size is proportional to their frequency of use. Their location in the evolutionary timeline (x-axis) depends on their naming in the oldest GO term. Their location on the y-axis depends on their precedence in the word string. For example, the oldest level 2 GO term “ion binding” (identifier GO:0043167) places the words “binding” and “ion” at age 0 of the timeline. The second oldest term “organic cyclic compound binding” (GO: 0097159) places the lexical units “compound” and “organic cyclic” at age 0.02. Similarly, the term “nucleoside-triphosphatase regulator activity” (GO:0060589) places the words “regulator” and “nucleoside-triphosphatase” at age 0.407 horizontally from the word “activity,” which appeared for the first time at age 0.068 with the term “transferase activity” (GO:0016740). If horizontal placement is impossible, the lexical unit is placed above its associated word for legibility.

Reading the word cloud from left to right and bottom to top shows a clear semantic evolutionary progression of the central lexical units of level 2 GO terms (Figure 6C). Most frequent words follow a temporal order: binding, activity, transporter, structural constituent, and channel. *Binding*, “the selective, non-covalent, often stoichiometric, interaction of a molecule with one or more specific sites on another molecule” (definition of level 1 GO term GO:523816), applied to “ions” (charged atoms or group of atoms), appears as the first molecular lexical unit. This function was soon coopted by other molecules, including organic cyclic and heterocyclic compounds, and then small molecules, closely followed by carbohydrate derivatives. Cooption continued throughout the timeline with binding of cofactors, drugs, and numerous Eukarya-specific lexical units associated with binding. Binding is a “communication” process that moves information from one agent to another, typically in 10 of Miller’s critical subsystems (Figure 3A). *Activity* is the second major lexical unit describing actions that occur at the molecular level. Its origin involves all six major Enzyme Commission (EC) categories of metabolic enzymes grouped according to the chemical reactions they catalyze: transferases, hydrolases, oxidoreductases, ligases, and lyases, in that order. These activities were followed by transporter activities first associated with membranes and transport specific to substrates and then with transcription factors, enzymes, channels, and neurotransmitters. Activities are “*action*” processes that move matter–energy in space–time, typically in eight of Miller’s critical subsystems. *Structural constituent* is the major Eukarya-specific lexical unit that originated with the eye lens, muscle, and myelin sheath and later unfolded with protein complexes, cytoskeleton, and cell wall. *Channel* is the major lexical unit shared by Eukarya and Bacteria, originally associated with “inhibitor” and “regulator” activities and part of the transmembrane “transport” system of the cell. The origination of all channel, transporter, and structural constituent lexical units emerged during the latter half of the timeline and are part of tangential Miller’s critical subsystems that provide transport, support, and storage to matter/energy and information flows.

Inferences derived from the timeline are consistent with previous work focusing on the history of molecular functions of structural domains in proteomes (Kim and Caetano-Anollés, 2010, Caetano-Anollés et al., 2011; Nasir and Caetano-Anollés, 2013; Nasir et al., 2014b; Kim et al., 2014; Caetano-Anollés et al., 2012). They support the early origin of metabolic activities through binding and catalysis (Koç and Caetano-Anollés, 2017), the early role of transferase and hydrolase activities (Pfeiffer et al., 2005) in a metabolic “big bang” (Caetano-Anollés et al., 2007), and the quick and massive development of biocatalytic mechanisms (Nath et al., 2014).

One remarkable observation that is supported by word clouds at other GO levels (data not shown) is the development of all central activities of the cell before age 0.5. These ancient activities are universal (ABE taxonomic group), while, with few exceptions (e.g., channel), the rest are Eukarya-specific and younger than 0.46. The very few words that were shared by two superkingdoms (AB, AE, and BE) were all younger than age 0.54, but most appeared after 0.8. These clear patterns are consistent with the pragmatic views of the triangle of persistence (Figure 3) and the existence of a saddle manifold that holds the last universal common ancestor and separates the ancestors of the two microbial superkingdoms from those of Eukarya. Since microbial interactions with the environment are constrained by low Reynolds numbers (R_*e*_ < 0.1) limiting fluid inertia (Purcell, 1977), this physical drag-delimited threshold likely splits life in the saddle into an economy-driven microbial world and a macroscopic world driven by mechanisms of flexibility and robustness (Yafremava et al., 2013; Mainzer et al., 2021). The word cloud of lexical units of level 2 terms now makes evident the functional activities associated with the two tendencies, one focusing on binding, enzymatic activities, transport, and regulation and the other on building higher-level structures with structural constituents and channels and regulatory and neurotransmitter activities.

## Conclusion and Prospects

Protein and nucleic acid macromolecules communicate by receiving and emitting information within the crowded environment of the cell, between cells in multicellular organisms, and with the environment. A number of methods in natural language processing have been used to discover functions in biological molecules that could explain this communication (Searls, 2002; Yandell and Majoros, 2002). This generally involves extracting molecular features with implementations that help analyze molecular functions with deep learning algorithms (Cai et al., 2009; Motomura et al., 2012; Asgari and Mofrad, 2015). For example, the use of artificial neural networks to represent protein sequences with dense *n*-dimensional vectors permitted to classify families of protein structures and intrinsic disorder with high accuracy (Asgari and Mofrad, 2015). What is missing is the use of this extracted information to understand languages established at scales of molecular organization higher than the protein sequence. Language laws that describe diversity, cohesion, and growth manifest in biological organization at modular unit, structure, function, and fitness levels. They suggest “evolutionary slowdowns” typical of economies of scale but use extant information to quantitatively derive recurrent statistical patterns of sublinear growth. Here, I propose that these patterns underlie a syntax, semantics, and pragmatics that can be mined with genomic information and advanced algorithmic implementations using the tools of the computational biologist. In doing so, I highlight the limitation of drawing analogies between language and biology. These analogies should be considered metaphors, even if language is a direct product of biology.

A reevaluation of comparative and evolutionary genomic data reveals a significant molecular and functional vocabulary compression in the organisms of microbial superkingdoms. This compression can be explained by the pragmatic framework of the triangle of persistence, which describes how molecular meaning is linked to molecular function and fitness in performance spaces. The economy-driven strategy of the triangle explains the push of the organisms of Bacteria and Archaea toward economy of molecular resources and smaller lexical, syntactic, and semantic repertoires. Since compression is the result of an evolutionary process (Krakauer, 2002), its measurement requires retrodiction. A kernel associated with the last universal common ancestor of life can be used to benchmark compression tendencies in diversified organisms. For example, the causal word cloud paradigm introduced in Figure 6C, which was inspired by the phylogenomic logo model of Caetano-Anollés and Seufferheld (2013), allows the easy visualization of how compression in prokaryotes restricted the development of flexibility and robustness mechanisms typical of Eukarya. Functional innovation of the microbial superkingdoms was restricted to a “kernel” of GO terms of molecular functions that are universally present in all three superkingdoms (the ABE Venn group) and were developed by the last universal ancestor of cellular life. While Eukarya unfolded significant innovations along the entire evolutionary timeline of GO terms, terms specific to the microbial superkingdoms were minimal if non-existent, while those specific to Eukarya dominated more recent history (Figures 5D, 6C). The evolutionary accumulation of GO terms informs about the evolutionary history of semantic and pragmatic compression. However, the possibility of indexing lexical units of GO terms with evolutionary ages quickly uncovers data-driven hypotheses that explain the evolutionary rise of complexity in biochemistry and the piecemeal evolution of proteomes and functionomes. I anticipate that the approach will be automated and further exploited by turning catena-inspired word clouds into time-driven network representations.

A number of “eukaryote signature proteins” (ESPs) involved in membrane remodeling and vesicle and cytoskeleton formation were identified in Asgardarchaeota, suggesting the likely existence of a Eukarya-like cytoskeleton in Archaea that could enable phagocytosis (Spang et al., 2015; Zaremba-Niedzwiedzka et al., 2017). These proteins together with phylogenetic trees reconstructed from concatenated sequence alignments of a small set of highly conserved genes have been used to propose an eocyte “two-domain” tree of life, challenging the classical three-superkingdom paradigm originally proposed by Carl R. Woese. However, ESPs are not exclusive of Asgards, since actin-related proteins have been detected in Bathyarchaeota (Zhou et al., 2018). This suggests that their presence in other phyla has been underestimated. In fact, a total 17 FSFs, which include domains in actin-depolymerizing and binding proteins, are shared between Eukarya and Asgard but are absent in other Archaea (Nasir et al., 2021). A substantial number of these FSFs (70%) are shared with Bacteria, including CPR bacteria (40%). These 17 FSFs have families that originated much later than the first Eukarya-specific and Archaea-specific families. This questions the proposal that Asgards and their EPSs are “missing links” bridging the gap between microbial superkingdoms and Eukarya. An expanded sampling of Asgard genomes concluded that ESPs are more likely the result of horizontal transfer, gene loss, and duplications (Liu et al., 2020). The analysis of the molecular and functional repertoires described in Figures 4–6 is again incompatible with the “two-domain” tree of life view. Protein domains and molecular functions exclusively shared by Archaea and Eukarya are a minority of recent evolutionary origin.

There is now a need to understand how interactions between organisms within and between superkingdoms are softening limitations of language compression through obligate parasitic and symbiotic interactions or by establishing collective behavior at microbial levels. For example, the development of bacterial multicellularity results in a multiplicity of phenotypic forms that have coopted strategies typical of Eukarya, including morphological differentiation, programmed cell death, and multicellular organized aggregation (patterning) (Claessen et al., 2014). The development of filaments, aggregations into biofilms and swarms, and multicellular magnetotactic assemblies may be triggered by physicochemical stress, nutrient scarcity, and environmental variability (Lyons and Koller, 2015). Multicellularity in Archaea is less understood but exists, for example, in acetate-utilizing *Methanosarcina* in the form of multicellular packets and lamina showing cell differentiation (Sowers et al., 1993). Cell aggregation of these archaeal microbes appears mediated by mechanical and adhesive properties facilitated by the S-layer methanochondroitin chains of the cell wall as well as turgor pressure (Milkevych et al., 2015). Remarkable interactions between organisms of Bacteria and Archaea exist that facilitate aggregations to enhance metabolic adaptations. Such is the interaction of *Methanosarcina* with biofilm-producing *Geobacteraceae* in iron-reducing environments (Zheng et al., 2015). These aggregations facilitate interspecies electron transfer between microbes of the two superkingdoms. These pragmatic pushes toward multicellularity are most likely late evolutionary adaptations that help microbes escape the economy-driven world of the persistence triangle. We may be witnessing the rise of new manifestations of diversity, cohesion, and growth in biological organization.

## Data Availability Statement

The original contributions presented in the study are included in the article/supplementary material, further inquiries can be directed to the corresponding author.

## Author Contributions

GC-A contributed to the design, experimentation, and analysis of the study and drafted, edited, improved, and finalized the manuscript.

## Conflict of Interest

The author declares that the research was conducted in the absence of any commercial or financial relationships that could be construed as a potential conflict of interest.
